# Crystal structure of (*E*)-1,3-dimethyl-2-[3-(3-nitro­phen­yl)triaz-2-en-1-yl­idene]-2,3-di­hydro-1*H*-imidazole

**DOI:** 10.1107/S1600536814020698

**Published:** 2014-09-20

**Authors:** Siddappa Patil, Alejandro Bugarin

**Affiliations:** a Department of Chemistry & Biochemistry, University of Texas at Arlington, PO Box, 19065, Arlington, TX 76019, USA

**Keywords:** crystal structure, azides, π-conjugated triazene, *N*-heterocyclic carbene, hydrogen bonds, π–π inter­actions

## Abstract

The title compound, C_11_H_12_N_6_O_2_, a π-conjugated triazene, crystallized with two independent mol­ecules (*A* and *B*) in the asymmetric unit. Both mol­ecules have an *E* conformation about the –N=N– bond and have slightly twisted overall conformations. In mol­ecule *A*, the imidazole ring is inclined to the benzene ring by 8.12 (4)°, while in mol­ecule *B* the two rings are inclined to one another by 7.73 (4)°. In the crystal, the independent mol­ecules are linked to each other by C—H⋯O hydrogen bonds, forming –*A*–*A*–*A*– and –*B*–*B*–*B–* chains along [100]. The chains are linked by C—H⋯O and C—H⋯N hydrogen bonds, forming sheets lying parallel to (001). The sheets are linked by further C—H⋯N hydrogen bonds and π–π inter­actions [centroid–centroid distance = 3.5243 (5) Å; involving the imidazole ring of mol­ecule A and the benzene ring of mol­ecule *B*], forming a three-dimensional framework structure.

## Abstract 2

The title mol­ecule, a rare example of a π-conjugated triazene, crystallized with two independent mol­ecules in the asymmetric unit. In the crystal, the two independent mol­ecules stack head-to-tail probably due to the presence of the dipole moment of the *meta* nitro group.

## Chemical context   

Triazenes are compounds containing three contiguous nitro­gen atoms in a linear format with a double bond between the first and second N atoms; *i.e*., –N=N—N–. The structure of the triazene moiety is influenced by the resonance arising from delocalization of the electron lone-pair on the third N atom, towards the double bond. Triazenes are relatively old compounds from the organic chemist’s viewpoint. It was as early as 1862 that Griess described a suitable method for the synthesis of 1,3-di­phenyl­triazene (Griess, 1862 ▶). At that time, no applications for triazenes could be found and these compounds were ignored for many decades. Unsubstituted triazenes are unstable under normal conditions; however, substituted triazenes are normally thermally stable. More recently, attention has been paid to substituted triazenes, especially to 1-aryl-3,3-dialkyl-triazenes [which were synthesized for the first time by Baeyer & Jaeger (1875 ▶)] because some of them show activity as insecticides (Giraldi *et al.*, 1990 ▶). Currently, triazenes have found uses as alkyl­ating agents in tumor therapy (Rouzer *et al.*, 1996 ▶), as iodo-masking groups in the synthesis of small (Nicolaou *et al.*, 1999 ▶) and macromolecules (Jones *et al.*, 1997 ▶), and in the preparation of *N*-containing heterocycles (Wirshun *et al.*, 1998 ▶). The first report on a π-conjugated triazenes was by Winberg *et al.* (1965 ▶), and more recently, we have reported the syntheses and structures of a variety of such π-conjugated triazenes (Patil *et al.*, 2014 ▶).
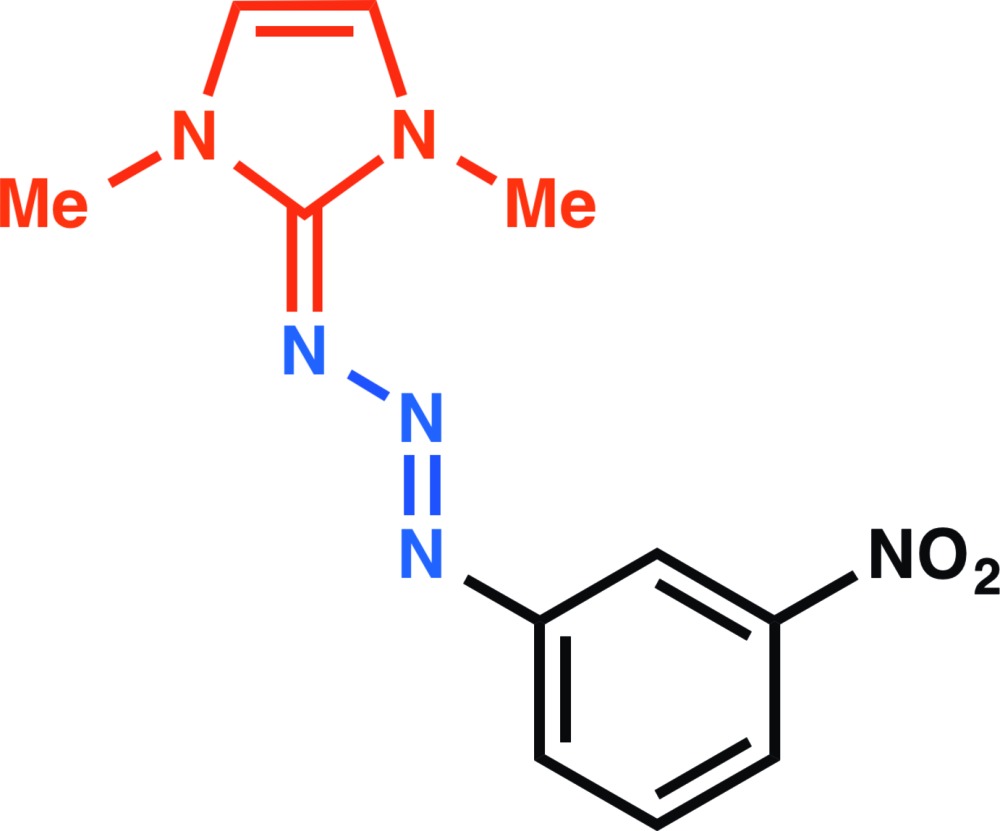


## Structural commentary   

**Figure 1:**
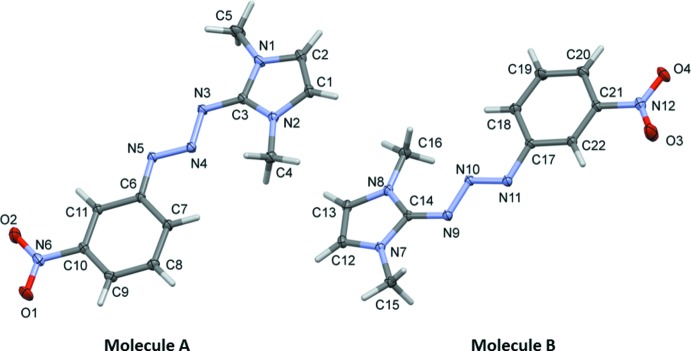
A view of the mol­ecular structure of the two independent mol­ecules (*A* and *B*) of the title compound, with atom labelling. Displacement ellipsoids are drawn at the 50% probability level.

The mol­ecular structures of the two independent mol­ecules (*A* and *B*) of the title compound are illustrated in Fig. 1 ▶. Both mol­ecules have an *E* conformation about the –N5=N4– and –N11=N10– bonds and the bond lengths and angles of the π-conjugated triazene unit (Table 1 ▶) are very similar to those in related structures (Khramov & Bielawski, 2005 ▶, 2007 ▶; Jishkariani *et al.*, 2013 ▶; Tennyson *et al.*, 2010 ▶). The two mol­ecules have slightly twisted overall conformations, with the imidazole ring (N1/N2/C1–C3) inclined to the benzene ring (C6–C11) by 8.12 (4)° in mol­ecule *A*, while in mol­ecule *B* the two rings (N7/N8/C12–C14 and C17–C22) are inclined to one another by 7.73 (4)°.

## Supra­molecular features   

**Figure 2:**
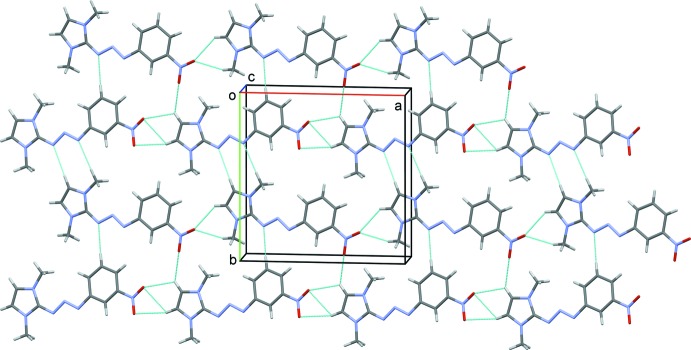
A view along the *c* axis of the crystal packing of title compound, with hydrogen bonds shown as dashed lines (see Table 2 ▶ for details).

**Figure 3:**
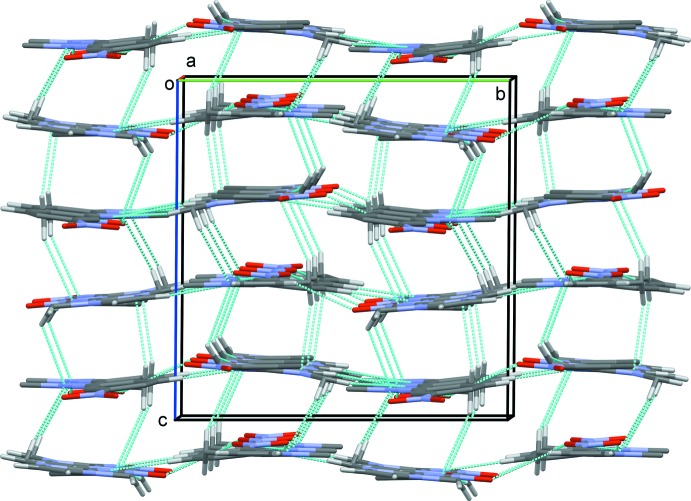
A view along the *a* axis of the crystal packing of the title compound, with hydrogen bonds shown as dashed lines (see Table 2 ▶ for details; H atoms not involved in hydrogen bonds have been omitted for clarity).

In the crystal, the independent mol­ecules are linked by C—H⋯O hydrogen bonds forming –*A*–*A*–*A*– and –*B*–*B*–*B*– chains along [100]. The chains are linked by C—H⋯O and C—H⋯N hydrogen bonds, forming sheets lying parallel to (001); see Fig. 2 ▶ and Table 2 ▶. The sheets are linked by further C—H⋯N hydrogen bonds and C—H⋯π and π–π inter­actions [*Cg*1⋯*Cg*4^i^ = 3.5243 (5) Å; *Cg*1 and *Cg*4 are the centroids of the imidazole ring of mol­ecule *A* and the benzene ring of mol­ecule *B*; symmetry code: (i) *x*, *y*, *z* − 1], forming a three-dimensional framework structure (Fig. 3 ▶ and Table 2 ▶).

## Database survey   

The first synthesis of a π-conjugated triazene was reported on in 1965 (Winberg & Coffman, 1965 ▶). The first X-ray structure analysis of a π-conjugated triazene appeared many years later (Khramov *et al.*, 2005 ▶). A search of the WebCSD database, gave 15 hits for π-conjugated triazenes. Two of these structures (Patil *et al.*, 2014 ▶) employed 1,3-di­methyl­imidazolium iodide as the carbene precursor. Although, there is a compound that closely resembles the title compound in the literature (Patil *et al.*, 2014 ▶), it differs in the position of the nitro-substituent in the aromatic moiety. In the title compound, the nitro substituent is in the *meta* position, while the parallel report has the nitro substituent in the *para* position.

## Synthesis and crystallization   

1-Azido-3-nitro­benzene was prepared according to the literature procedure (Siddiki *et al.*, 2013 ▶). The synthesis of 1,3-di­methyl­imidazolium iodide was carried out accordingly to literature procedure (Oertel *et al.*, 2011 ▶). For the synthesis of the title compound, 1-azido-3-nitro­benzene (196 mg, 1.2 mmol) was added in one portion to a suspension of 1,3-di­methyl­imidazolium iodide (134 mg, 0.6 mmol) in dry THF (5 mL) and stirred at room temperature for 5 min. In one portion, NaH (24 mg, 0.6 mmol, 60% in mineral oil) was added to the reaction vessel and the resulting mixture was stirred at room temperature for 6 h. The yellowish-orange precipitate that formed was collected by filtration and dried under reduced pressure, giving the title compound as an orange crystalline solid (yield 140 mg, 90%). Crystals were prepared by slow infusion of hexa­nes into a saturated THF solution of the title compound. IR (neat) ν 3439, 1601, 1398, 1357, 1191 cm^−1. 1^H NMR (500 MHz, DMSO-*d*_6_): δ 7.99 (*s*, 1H, Ph-H), 7.85–7.83 (*m*, 1H, Ph-H), 7.70–7.69 (*m*, 1 H, Ph-H), 7.55–7.52 (*m*, 1H, Ph-H), 7.06 (*s*, 2H, NCH) 3.60 (*s*, 6H, N-CH_3_). ^13^C NMR (125 MHz, DMSO-*d*_6_): δ 154.4, 151.1, 149.0, 130.6, 126.9, 118.8, 118.3, 114.4, 35.7. UV/Vis (0.1 µ*M*, CH_2_Cl_2_): λ (∊) = 455 nm. HRMS (ESI, N_2_): *m*/*z* calculated for C_11_H_13_N_6_O_2_ [*M* + H]^+^ 261.1095, found 261.1094.

## Refinement   

Crystal data, data collection and structure refinement details are summarized in Table 3 ▶. The C-bound H atoms were included in calculated positions and treated as riding atoms: C-H = 0.95 and 0.98 Å for CH and CH_3_ H atoms, respectively, with U_iso_(H) = 1.5U_eq_(C) for methyl H atoms and = 1.2U_eq_(C) for other H atoms.

## Supplementary Material

Crystal structure: contains datablock(s) I. DOI: 10.1107/S1600536814020698/su2778sup1.cif

Structure factors: contains datablock(s) I. DOI: 10.1107/S1600536814020698/su2778Isup2.hkl

Supporting information file. DOI: 10.1107/S1600536814020698/su2778Isup3.cml

CCDC reference: 977732

Additional supporting information:  crystallographic information; 3D view; checkCIF report

## Figures and Tables

**Table 1:** Selected geometric parameters (Å, °)

N3—C3	1.3532 (9)	N9—C14	1.3501 (9)
N3—N4	1.3318 (8)	N9—N10	1.3299 (8)
N4—N5	1.2856 (8)	N10—N11	1.2866 (8)
			
N4—N3—C3	112.23 (6)	N10—N9—C14	112.44 (6)
N5—N4—N3	111.84 (6)	N11—N10—N9	111.74 (6)
N4—N5—C6	111.86 (6)	N10—N11—C17	111.77 (6)

**Table 2:** Hydrogen-bond geometry (Å, °) *Cg*2 and *Cg*3 are the centroids of the benzene ring (C6–C11) of mol­ecule *A* and the imidazole ring (N7/N8/C12–C14) ring of mol­ecule *B*, respectively.

*D*—H⋯*A*	*D*—H	H⋯*A*	*D*⋯*A*	*D*—H⋯*A*
C1—H1⋯O3^i^	0.95	2.55	3.3223 (11)	139
C16—H00*B*⋯N5^ii^	0.98	2.50	3.4757 (11)	172
C16—H00*C*⋯N3^iii^	0.98	2.61	3.5557 (11)	163
C8—H8⋯N9^iv^	0.95	2.44	3.3882 (10)	178
C13—H13⋯N3^ii^	0.95	2.60	3.5441 (10)	174
C15—H15*B*⋯O4^v^	0.98	2.48	3.3692 (11)	151
C4—H4*C*⋯*Cg*3^vi^	0.98	2.96	3.8391 (9)	150
C15—H15*A*⋯*Cg*2^iii^	0.98	2.80	3.5398 (9)	132

Symmetry codes: (i) 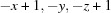; (ii) 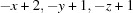; (iii) 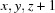; (iv) 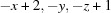; (v) 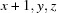; (vi) 
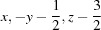
.

**Table 3:** Experimental details

Crystal data	
Chemical formula	C_11_H_12_N_6_O_2_
*M*_r_	260.27
Crystal system, space group	Monoclinic, *P*2_1_/*c*
Temperature (K)	103
*a*, *b*, *c* (Å)	14.0377 (5), 12.9071 (5), 14.2995 (5)
β (°)	113.6050 (8)
*V* (Å^3^)	2374.08 (15)
*Z*	8
Radiation type	Mo *K*α
μ (mm^−1^)	0.11
Crystal size (mm)	0.43 × 0.33 × 0.25

Data collection	
Diffractometer	Bruker SMART APEXII
Absorption correction	Multi-scan (*SADABS*; Bruker, 2014 ▶)
*T*_min_, *T*_max_	0.952, 1.000
No. of measured, independent and observed [*I* > 2σ(*I*)] reflections	60704, 14895, 10565
*R*_int_	0.040
(sin θ/λ)_max_ (Å^−1^)	0.916

Refinement	
*R*[*F* ^2^ > 2σ(*F* ^2^)], *wR*(*F* ^2^), *S*	0.049, 0.128, 1.03
No. of reflections	14895
No. of parameters	347
H-atom treatment	H-atom parameters constrained
Δρ_max_, Δρ_min_ (e Å^−3^)	0.69, −0.30

Computer programs: *APEX2* and *SAINT* (Bruker, 2014 ▶), *SHELXS97* and *SHELXL2013* (Sheldrick 2008 ▶), *Mercury* (Macrae *et al.*, 2008 ▶), *PLATON* (Spek, 2009 ▶) and *publCIF* (Westrip, 2010 ▶).

## supplementary crystallographic information

### Crystal data

**Table:**

C_11_H_12_N_6_O_2_	*F*(000) = 1088
*M**_r_* = 260.27	*D*_x_ = 1.456 Mg m^−^^3^
Monoclinic, *P*2_1_/*c*	Mo *K*α radiation, λ = 0.71073 Å
*a* = 14.0377 (5) Å	Cell parameters from 9007 reflections
*b* = 12.9071 (5) Å	θ = 3.1–40.1°
*c* = 14.2995 (5) Å	µ = 0.11 mm^−^^1^
β = 113.6050 (8)°	*T* = 103 K
*V* = 2374.08 (15) Å^3^	Prism, orange
*Z* = 8	0.43 × 0.33 × 0.25 mm

### Data collection

**Table:**

Bruker SMART APEXII diffractometer	10565 reflections with *I* > 2σ(*I*)
φ and ω scans	*R*_int_ = 0.040
Absorption correction: multi-scan (*SADABS*; Bruker, 2014)	θ_max_ = 40.7°, θ_min_ = 2.9°
*T*_min_ = 0.952, *T*_max_ = 1.000	*h* = −25→25
60704 measured reflections	*k* = −23→23
14895 independent reflections	*l* = −26→26

### Refinement

**Table:**

Refinement on *F*^2^	Primary atom site location: structure-invariant direct methods
Least-squares matrix: full	Secondary atom site location: difference Fourier map
*R*[*F*^2^ > 2σ(*F*^2^)] = 0.049	Hydrogen site location: inferred from neighbouring sites
*wR*(*F*^2^) = 0.128	H-atom parameters constrained
*S* = 1.03	*w* = 1/[σ^2^(*F*_o_^2^) + (0.060*P*)^2^ + 0.4222*P*] where *P* = (*F*_o_^2^ + 2*F*_c_^2^)/3
14895 reflections	(Δ/σ)_max_ = 0.001
347 parameters	Δρ_max_ = 0.69 e Å^−^^3^
0 restraints	Δρ_min_ = −0.30 e Å^−^^3^

### Special details

**Table:**

Geometry. All e.s.d.'s (except the e.s.d. in the dihedral angle between two l.s. planes) are estimated using the full covariance matrix. The cell e.s.d.'s are taken into account individually in the estimation of e.s.d.'s in distances, angles and torsion angles; correlations between e.s.d.'s in cell parameters are only used when they are defined by crystal symmetry. An approximate (isotropic) treatment of cell e.s.d.'s is used for estimating e.s.d.'s involving l.s. planes.

### Fractional atomic coordinates and isotropic or equivalent isotropic displacement parameters (Å^2^)

**Table:**

	*x*	*y*	*z*	*U*_iso_*/*U*_eq_	
O1	1.41154 (5)	0.17070 (6)	0.07592 (6)	0.02761 (14)	
O2	1.35616 (5)	0.32803 (5)	0.07112 (6)	0.02894 (15)	
N1	0.69882 (5)	0.35407 (5)	0.09704 (5)	0.01430 (10)	
N2	0.74421 (5)	0.19273 (5)	0.09668 (5)	0.01287 (10)	
N3	0.86553 (5)	0.33461 (5)	0.09907 (5)	0.01281 (10)	
N4	0.93463 (4)	0.26313 (5)	0.10078 (4)	0.01161 (9)	
N5	1.01715 (5)	0.30405 (5)	0.09846 (5)	0.01286 (10)	
N6	1.34736 (5)	0.23387 (6)	0.07898 (5)	0.01852 (12)	
C1	0.64415 (5)	0.19428 (6)	0.09429 (6)	0.01542 (12)	
H1	0.6029	0.1354	0.0927	0.019*	
C2	0.61618 (5)	0.29407 (6)	0.09470 (6)	0.01605 (12)	
H2	0.5519	0.3186	0.0936	0.019*	
C3	0.77707 (5)	0.29211 (5)	0.09821 (5)	0.01153 (10)	
C4	0.79822 (6)	0.09749 (6)	0.09254 (6)	0.01641 (12)	
H4A	0.8149	0.0987	0.0322	0.025*	
H4B	0.7535	0.0379	0.0884	0.025*	
H4C	0.8626	0.0918	0.1542	0.025*	
C5	0.70332 (7)	0.46623 (6)	0.09997 (8)	0.02396 (16)	
H5A	0.7448	0.4892	0.1699	0.036*	
H5B	0.6328	0.4943	0.0774	0.036*	
H5C	0.7354	0.4910	0.0546	0.036*	
C6	1.09080 (5)	0.22826 (5)	0.10102 (5)	0.01144 (10)	
C7	1.08081 (6)	0.12097 (5)	0.11242 (6)	0.01449 (11)	
H7	1.0200	0.0949	0.1180	0.017*	
C8	1.15869 (6)	0.05259 (6)	0.11562 (6)	0.01589 (12)	
H8	1.1512	−0.0194	0.1249	0.019*	
C9	1.24754 (6)	0.08873 (6)	0.10535 (6)	0.01545 (12)	
H9	1.3009	0.0426	0.1071	0.019*	
C10	1.25535 (5)	0.19462 (6)	0.09241 (5)	0.01371 (11)	
C11	1.18020 (5)	0.26503 (5)	0.09144 (5)	0.01285 (11)	
H11	1.1893	0.3371	0.0844	0.015*	
O3	0.37570 (6)	0.04437 (6)	0.82832 (7)	0.03536 (18)	
O4	0.28471 (5)	0.17434 (6)	0.84106 (6)	0.02731 (14)	
N7	1.02372 (4)	0.23808 (5)	0.84318 (5)	0.01204 (10)	
N8	0.94498 (5)	0.37758 (5)	0.86112 (5)	0.01262 (10)	
N9	0.86200 (5)	0.20437 (5)	0.84779 (5)	0.01280 (10)	
N10	0.77639 (4)	0.25054 (5)	0.84548 (5)	0.01187 (9)	
N11	0.70763 (5)	0.18431 (5)	0.84484 (5)	0.01359 (10)	
N12	0.36319 (5)	0.13767 (6)	0.83579 (6)	0.01928 (12)	
C12	1.08846 (5)	0.32082 (6)	0.84914 (6)	0.01436 (11)	
H12	1.1547	0.3174	0.8459	0.017*	
C13	1.03999 (6)	0.40748 (6)	0.86051 (6)	0.01455 (12)	
H13	1.0660	0.4762	0.8669	0.017*	
C14	0.93575 (5)	0.27292 (5)	0.85061 (5)	0.01114 (10)	
C15	1.04585 (5)	0.12916 (6)	0.83482 (6)	0.01494 (12)	
H15A	1.0610	0.0946	0.9003	0.022*	
H15B	1.1061	0.1231	0.8169	0.022*	
H15C	0.9854	0.0964	0.7817	0.022*	
C16	0.87328 (6)	0.45063 (6)	0.87669 (7)	0.01784 (13)	
H00A	0.8088	0.4537	0.8149	0.027*	
H00B	0.9052	0.5195	0.8912	0.027*	
H00C	0.8576	0.4277	0.9343	0.027*	
C17	0.61624 (5)	0.23313 (5)	0.84061 (5)	0.01230 (11)	
C18	0.59905 (6)	0.34078 (6)	0.83778 (6)	0.01640 (12)	
H18	0.6516	0.3870	0.8374	0.020*	
C19	0.50599 (6)	0.37999 (6)	0.83552 (7)	0.01905 (14)	
H19	0.4958	0.4528	0.8338	0.023*	
C20	0.42745 (6)	0.31436 (6)	0.83575 (6)	0.01717 (13)	
H20	0.3641	0.3410	0.8352	0.021*	
C21	0.44519 (5)	0.20858 (6)	0.83684 (6)	0.01418 (11)	
C22	0.53703 (5)	0.16660 (6)	0.83938 (6)	0.01394 (11)	
H22	0.5461	0.0936	0.8403	0.017*	

### Atomic displacement parameters (Å^2^)

**Table:**

	*U*^11^	*U*^22^	*U*^33^	*U*^12^	*U*^13^	*U*^23^
O1	0.0170 (3)	0.0305 (3)	0.0415 (4)	0.0060 (2)	0.0181 (3)	0.0032 (3)
O2	0.0208 (3)	0.0227 (3)	0.0491 (4)	−0.0023 (2)	0.0200 (3)	0.0063 (3)
N1	0.0111 (2)	0.0138 (2)	0.0193 (3)	0.00077 (18)	0.0074 (2)	−0.0021 (2)
N2	0.0114 (2)	0.0121 (2)	0.0160 (2)	−0.00117 (18)	0.00653 (19)	−0.00044 (19)
N3	0.0110 (2)	0.0111 (2)	0.0178 (3)	0.00034 (17)	0.00737 (19)	−0.00037 (19)
N4	0.0106 (2)	0.0111 (2)	0.0141 (2)	0.00008 (17)	0.00600 (18)	−0.00066 (18)
N5	0.0115 (2)	0.0108 (2)	0.0184 (3)	0.00012 (17)	0.0082 (2)	0.00033 (19)
N6	0.0117 (2)	0.0232 (3)	0.0219 (3)	0.0010 (2)	0.0081 (2)	0.0025 (2)
C1	0.0117 (3)	0.0181 (3)	0.0177 (3)	−0.0026 (2)	0.0071 (2)	−0.0003 (2)
C2	0.0112 (3)	0.0201 (3)	0.0183 (3)	−0.0011 (2)	0.0074 (2)	−0.0017 (2)
C3	0.0104 (2)	0.0118 (2)	0.0129 (3)	−0.00022 (19)	0.0053 (2)	−0.0012 (2)
C4	0.0168 (3)	0.0109 (3)	0.0226 (3)	−0.0004 (2)	0.0091 (3)	−0.0003 (2)
C5	0.0183 (3)	0.0141 (3)	0.0405 (5)	0.0018 (2)	0.0129 (3)	−0.0054 (3)
C6	0.0114 (2)	0.0105 (2)	0.0133 (3)	0.00012 (19)	0.0059 (2)	−0.0003 (2)
C7	0.0151 (3)	0.0110 (2)	0.0198 (3)	−0.0003 (2)	0.0096 (2)	−0.0001 (2)
C8	0.0169 (3)	0.0111 (3)	0.0206 (3)	0.0013 (2)	0.0085 (2)	−0.0004 (2)
C9	0.0134 (3)	0.0147 (3)	0.0181 (3)	0.0027 (2)	0.0062 (2)	−0.0014 (2)
C10	0.0104 (2)	0.0160 (3)	0.0156 (3)	0.0001 (2)	0.0061 (2)	−0.0002 (2)
C11	0.0114 (2)	0.0127 (3)	0.0154 (3)	−0.0003 (2)	0.0063 (2)	0.0004 (2)
O3	0.0258 (3)	0.0183 (3)	0.0696 (6)	−0.0036 (2)	0.0271 (4)	0.0009 (3)
O4	0.0134 (2)	0.0315 (3)	0.0412 (4)	0.0020 (2)	0.0153 (3)	0.0029 (3)
N7	0.0102 (2)	0.0114 (2)	0.0153 (2)	−0.00043 (17)	0.00589 (18)	−0.00002 (18)
N8	0.0132 (2)	0.0096 (2)	0.0164 (2)	−0.00070 (18)	0.00730 (19)	0.00062 (18)
N9	0.0106 (2)	0.0106 (2)	0.0190 (3)	−0.00018 (17)	0.00774 (19)	0.00041 (19)
N10	0.0105 (2)	0.0119 (2)	0.0140 (2)	0.00009 (17)	0.00575 (18)	0.00015 (18)
N11	0.0114 (2)	0.0120 (2)	0.0194 (3)	0.00001 (18)	0.0083 (2)	0.00108 (19)
N12	0.0125 (2)	0.0210 (3)	0.0260 (3)	−0.0008 (2)	0.0093 (2)	0.0023 (2)
C12	0.0117 (3)	0.0150 (3)	0.0172 (3)	−0.0023 (2)	0.0067 (2)	0.0013 (2)
C13	0.0139 (3)	0.0131 (3)	0.0172 (3)	−0.0029 (2)	0.0068 (2)	0.0010 (2)
C14	0.0105 (2)	0.0104 (2)	0.0130 (3)	−0.00021 (19)	0.0052 (2)	0.00045 (19)
C15	0.0134 (3)	0.0124 (3)	0.0200 (3)	0.0021 (2)	0.0077 (2)	0.0006 (2)
C16	0.0198 (3)	0.0102 (3)	0.0278 (4)	0.0011 (2)	0.0140 (3)	−0.0004 (2)
C17	0.0108 (2)	0.0124 (3)	0.0145 (3)	0.00085 (19)	0.0058 (2)	0.0005 (2)
C18	0.0150 (3)	0.0124 (3)	0.0237 (3)	0.0004 (2)	0.0098 (3)	0.0003 (2)
C19	0.0167 (3)	0.0134 (3)	0.0289 (4)	0.0030 (2)	0.0111 (3)	0.0004 (3)
C20	0.0131 (3)	0.0173 (3)	0.0222 (3)	0.0029 (2)	0.0082 (2)	0.0001 (2)
C21	0.0105 (2)	0.0157 (3)	0.0172 (3)	−0.0002 (2)	0.0064 (2)	0.0004 (2)
C22	0.0115 (2)	0.0131 (3)	0.0184 (3)	0.0006 (2)	0.0071 (2)	0.0010 (2)

### Geometric parameters (Å, º)

**Table:**

O1—N6	1.2288 (9)	O3—N12	1.2278 (10)
O2—N6	1.2313 (10)	O4—N12	1.2287 (9)
N1—C3	1.3534 (9)	N7—C14	1.3578 (9)
N1—C2	1.3841 (9)	N7—C12	1.3827 (9)
N1—C5	1.4489 (10)	N7—C15	1.4549 (9)
N2—C3	1.3603 (9)	N8—C14	1.3597 (9)
N2—C1	1.3914 (9)	N8—C13	1.3918 (9)
N2—C4	1.4578 (9)	N8—C16	1.4598 (9)
N3—C3	1.3532 (9)	N9—C14	1.3501 (9)
N3—N4	1.3318 (8)	N9—N10	1.3299 (8)
N4—N5	1.2856 (8)	N10—N11	1.2866 (8)
N5—C6	1.4132 (9)	N11—C17	1.4087 (9)
N6—C10	1.4699 (9)	N12—C21	1.4659 (10)
C1—C2	1.3473 (11)	C12—C13	1.3525 (10)
C1—H1	0.9500	C12—H12	0.9500
C2—H2	0.9500	C13—H13	0.9500
C4—H4A	0.9800	C15—H15A	0.9800
C4—H4B	0.9800	C15—H15B	0.9800
C4—H4C	0.9800	C15—H15C	0.9800
C5—H5A	0.9800	C16—H00A	0.9800
C5—H5B	0.9800	C16—H00B	0.9800
C5—H5C	0.9800	C16—H00C	0.9800
C6—C11	1.3987 (9)	C17—C22	1.3994 (10)
C6—C7	1.4078 (10)	C17—C18	1.4081 (10)
C7—C8	1.3916 (10)	C18—C19	1.3893 (10)
C7—H7	0.9500	C18—H18	0.9500
C8—C9	1.3934 (10)	C19—C20	1.3913 (11)
C8—H8	0.9500	C19—H19	0.9500
C9—C10	1.3893 (10)	C20—C21	1.3868 (11)
C9—H9	0.9500	C20—H20	0.9500
C10—C11	1.3884 (10)	C21—C22	1.3855 (10)
C11—H11	0.9500	C22—H22	0.9500
			
C3—N1—C2	109.75 (6)	C14—N7—C12	109.60 (6)
C3—N1—C5	124.35 (6)	C14—N7—C15	123.86 (6)
C2—N1—C5	125.89 (6)	C12—N7—C15	126.49 (6)
C3—N2—C1	108.62 (6)	C14—N8—C13	108.90 (6)
C3—N2—C4	128.12 (6)	C14—N8—C16	128.14 (6)
C1—N2—C4	123.19 (6)	C13—N8—C16	122.88 (6)
N4—N3—C3	112.23 (6)	N10—N9—C14	112.44 (6)
N5—N4—N3	111.84 (6)	N11—N10—N9	111.74 (6)
N4—N5—C6	111.86 (6)	N10—N11—C17	111.77 (6)
O1—N6—O2	123.36 (7)	O3—N12—O4	123.10 (7)
O1—N6—C10	118.10 (7)	O3—N12—C21	118.31 (6)
O2—N6—C10	118.54 (7)	O4—N12—C21	118.59 (7)
C2—C1—N2	107.86 (6)	C13—C12—N7	107.23 (6)
C2—C1—H1	126.1	C13—C12—H12	126.4
N2—C1—H1	126.1	N7—C12—H12	126.4
C1—C2—N1	106.98 (6)	C12—C13—N8	107.52 (6)
C1—C2—H2	126.5	C12—C13—H13	126.2
N1—C2—H2	126.5	N8—C13—H13	126.2
N1—C3—N3	119.86 (6)	N9—C14—N7	119.42 (6)
N1—C3—N2	106.78 (6)	N9—C14—N8	133.82 (6)
N3—C3—N2	133.35 (6)	N7—C14—N8	106.75 (6)
N2—C4—H4A	109.5	N7—C15—H15A	109.5
N2—C4—H4B	109.5	N7—C15—H15B	109.5
H4A—C4—H4B	109.5	H15A—C15—H15B	109.5
N2—C4—H4C	109.5	N7—C15—H15C	109.5
H4A—C4—H4C	109.5	H15A—C15—H15C	109.5
H4B—C4—H4C	109.5	H15B—C15—H15C	109.5
N1—C5—H5A	109.5	N8—C16—H00A	109.5
N1—C5—H5B	109.5	N8—C16—H00B	109.5
H5A—C5—H5B	109.5	H00A—C16—H00B	109.5
N1—C5—H5C	109.5	N8—C16—H00C	109.5
H5A—C5—H5C	109.5	H00A—C16—H00C	109.5
H5B—C5—H5C	109.5	H00B—C16—H00C	109.5
C11—C6—C7	118.69 (6)	C22—C17—C18	118.63 (6)
C11—C6—N5	115.99 (6)	C22—C17—N11	115.56 (6)
C7—C6—N5	125.31 (6)	C18—C17—N11	125.81 (6)
C8—C7—C6	120.95 (6)	C19—C18—C17	120.58 (7)
C8—C7—H7	119.5	C19—C18—H18	119.7
C6—C7—H7	119.5	C17—C18—H18	119.7
C7—C8—C9	120.58 (7)	C18—C19—C20	121.12 (7)
C7—C8—H8	119.7	C18—C19—H19	119.4
C9—C8—H8	119.7	C20—C19—H19	119.4
C10—C9—C8	117.72 (6)	C21—C20—C19	117.42 (7)
C10—C9—H9	121.1	C21—C20—H20	121.3
C8—C9—H9	121.1	C19—C20—H20	121.3
C11—C10—C9	123.03 (6)	C22—C21—C20	123.12 (7)
C11—C10—N6	118.49 (6)	C22—C21—N12	118.34 (6)
C9—C10—N6	118.48 (6)	C20—C21—N12	118.55 (6)
C10—C11—C6	119.00 (6)	C21—C22—C17	119.12 (6)
C10—C11—H11	120.5	C21—C22—H22	120.4
C6—C11—H11	120.5	C17—C22—H22	120.4

### Hydrogen-bond geometry (Å, º)

Cg2 and Cg3 are the centroids of the benzene ring (C6–C11) of molecule *A* and the imidazole ring (N7/N8/C12–C14) ring of molecule *B*, respectively.

**Table:**

*D*—H···*A*	*D*—H	H···*A*	*D*···*A*	*D*—H···*A*
C1—H1···O3^i^	0.95	2.55	3.3223 (11)	139
C16—H00*B*···N5^ii^	0.98	2.50	3.4757 (11)	172
C16—H00*C*···N3^iii^	0.98	2.61	3.5557 (11)	163
C8—H8···N9^iv^	0.95	2.44	3.3882 (10)	178
C13—H13···N3^ii^	0.95	2.60	3.5441 (10)	174
C15—H15*B*···O4^v^	0.98	2.48	3.3692 (11)	151
C4—H4*C*···*Cg*3^vi^	0.98	2.96	3.8391 (9)	150
C15—H15*A*···*Cg*2^iii^	0.98	2.80	3.5398 (9)	132

Symmetry codes: (i) −*x*+1, −*y*, −*z*+1; (ii) −*x*+2, −*y*+1, −*z*+1; (iii) *x*, *y*, *z*+1; (iv) −*x*+2, −*y*, −*z*+1; (v) *x*+1, *y*, *z*; (vi) *x*, −*y*−1/2, *z*−3/2.
